# Effects of ozone therapy on facial nerve regeneration^☆^

**DOI:** 10.1016/j.bjorl.2016.02.009

**Published:** 2016-04-22

**Authors:** Isa Ozbay, Ilker Ital, Cuneyt Kucur, Raziye Akcılar, Aysenur Deger, Savas Aktas, Fatih Oghan

**Affiliations:** a Dumlupinar University, Department of Otolaryngology, Kutahya, Turkey; b Dumlupinar University, Department of Anesthesiology and Reanimation, Kutahya, Turkey; c Dumlupinar University, Department of Physiology, Kutahya, Turkey; d Dumlupinar University, Department of Pathology, Kutahya, Turkey; e Mersin University, Department of Histology and Embryology, Mersin, Turkey

**Keywords:** Ozone, Regeneration, Facial nerve, Ozônio, Regeneração, Nervo facial

## Abstract

**Introduction:**

Ozone may promote moderate oxidative stress, which increases antioxidant endogenous systems. There are a number of antioxidants that have been investigated therapeutically for improving peripheral nerve regeneration. However, no previous studies have reported the effect of ozone therapy on facial nerve regeneration.

**Objective:**

We aimed to evaluate the effect of ozone therapy on facial nerve regeneration.

**Methods:**

Fourteen Wistar albino rats were randomly divided into two groups with experimental nerve crush injuries: a control group, which received saline treatment post-crush, and an experimental group, which received ozone treatment. All animals underwent surgery in which the left facial nerve was exposed and crushed. Treatment with saline or ozone began on the day of the nerve crush. Left facial nerve stimulation thresholds were measured before crush, immediately after crush, and after 30 days. After measuring nerve stimulation thresholds at 30 days post-injury, the crushed facial nerve was excised. All specimens were studied using light and electron microscopy.

**Results:**

Post-crushing, the ozone-treated group had lower stimulation thresholds than the saline group. Although this did not achieve statistical significance, it is indicative of greater functional improvement in the ozone group. Significant differences were found in vascular congestion, macrovacuolization, and myelin thickness between the ozone and control groups. Significant differences were also found in axonal degeneration and myelin ultrastructure between the two groups.

**Conclusion:**

We found that ozone therapy exerted beneficial effect on the regeneration of crushed facial nerves in rats.

## Resumo

**Introdução:**

O ozônio pode promover estresse oxidativo moderado, o que aumenta sistemas endógenos antioxidantes. Há determinado número de antioxidantes sendo investigados terapeuticamente para melhorar a regeneração do nervo periférico. No entanto, nenhum estudo anterior relatou o efeito da terapia com ozônio na regeneração do nervo facial.

**Objetivo:**

Nosso objetivo foi avaliar o efeito da terapia com ozônio na regeneração do nervo facial.

**Método:**

Ao todo, 14 ratos albinos Wistar foram divididos aleatoriamente em dois grupos com lesões experimentais por esmagamento do nervo: um grupo controle, que recebeu tratamento com solução salina pós-esmagamento; e um grupo experimental, que recebeu tratamento com ozônio. Todos os animais foram submetidos a cirurgia na qual o nervo facial esquerdo foi exposto e esmagado. O tratamento com solução salina ou ozônio se iniciou no dia do esmagamento do nervo. Os limiares de estimulação do nervo facial esquerdo foram medidos antes do esmagamento, imediatamente após o esmagamento e após 30 dias. Depois de medir limiares de estimulação do nervo aos 30 dias pós-lesão, o nervo facial esmagado foi excisado. Todas as amostras foram estudadas por meio de microscopia óptica e eletrônica.

**Resultados:**

Após o esmagamento, o grupo tratado com ozônio apresentou menores limiares de estimulação do que o grupo da solução salina. Embora isto não tenha significância estatística, é indicativo de maior melhora funcional no grupo do ozônio. Foram encontradas diferenças significativas na congestão vascular, macrovacuolização e espessura da mielina entre os grupos do ozônio e controle. Diferenças significativas também foram encontradas na degeneração axonal e ultraestrutura de mielina entre os dois grupos.

**Conclusão:**

Verificou-se que a terapia com ozônio teve efeito benéfico sobre a regeneração dos nervos faciais esmagados em ratos.

## Introduction

Peripheral facial palsy is the most frequent cranial neuropathy and may arise from diverse mechanisms of injury to the seventh cranial nerve. After injury, regeneration of the facial nerve is problematic. Nerve injury, such as lipid peroxidation of neurovascular cells, can lead to oxidative stress as a result of the production of free radicals.^1^, ^2^ Various methods have been used to enhance peripheral nerve regeneration.^3^, ^4^ It is well known that oxygen free radicals influence nerve regeneration, and additionally, some studies have demonstrated that antioxidants reduce the levels of free oxygen radicals.^5^, ^6^

Ozone (O_3_), a powerful oxidant, is non-persistent with a half-life of approximately 20 min at normal temperatures.^7^ It decomposes and disperses in water easily. O_3_ can restrain inflammatory cell factors, activate cyclooxygenase, and decrease the stress reaction to histiocytic oxidation, augmenting the histiocytic ability of resisting oxidation and free radicals.^7^ It can also scavenge the free radicals resulting from chronic inflammation, can serve as a painkiller and is anti-inflammatory.^8^

The concept of using ozone to improve the healing of infected wounds, necrotic, or poorly oxygenated tissue has been explored in orthopedics, dentistry and with skin wounds.^9^ However, no previous study has reported on the effect of ozone therapy on facial nerve regeneration. Therefore, we investigated the effect of ozone therapy on facial nerve regeneration in rats. To the best of our knowledge, this is the first study to evaluate ozone therapy in this context.

## Methods

### Study design

Fourteen Wistar albino rats with a mean (SD) weight of 250–300 g were housed in groups for 7–14 days under standard environmental conditions, with free access to food and water. The rats were randomly divided into two groups identified as control and ozone: the ozone group (*n* = 7) received an ozone dose of 1.1 mg/kg/d intraperitoneal (IP) for 30 days, and the controls (*n* = 7) received 1.1 mg/kg/d IP of saline for 30 days. All animal procedures were performed in accordance with the European Communities Council Directive of 1986 and with approval gained by the local Animal Ethics Committee (2015.02.03).

### Facial nerve crush

**Figure 1:**
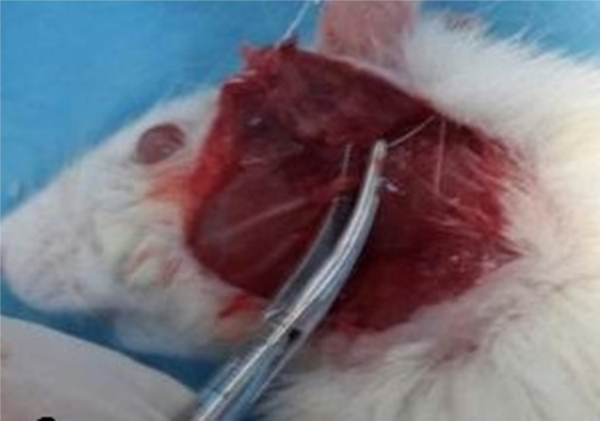
Facial nerve crush surgery. The truncus of the facial nerve was dissected from the adjacent tissue and was then placed between two pairs of hemostatic mosquito clamps.

Rats were sedated with IP injections of ketamine (80 mg/kg) and xylazine (5 mg/kg). Skin over the left facial nerve was shaved and cleaned with iodine. The left facial nerve was exposed with an oblique incision inferior to the auricule, with visual identification of the main trunk of the facial nerve (Fig. 1). The facial nerve trunk was then crushed for 1 min with a hemostatic mosquito clamp at the first level without cutting the axon.

### Ozone application

Ozone was generated with an ozone generator (Humazon^®^ ProMedic-Humares GmbH, Germany). The O_3_ flow rate was kept constant at 3 L/min, representing a concentration of 50 μg/mL and approximately 3% of the O_3_/O_2_ gas mixture. Ozone resistant Tygon polymer tubes and single-use silicon treated polypropylene were used throughout the experiment to ensure containment of O_3_ and consistency of concentrations. The ozone given to each animal was adjusted to a final dose of 1.1 mg/kg (1) and was given IP once daily for 30 days.

### Electrophysiological threshold assessment

The facial nerve stimulation threshold was measured using a Nerve Integrity Monitor (NIM-2; Medtronic Xomed, Jacksonville, FL). Before crushing the nerve, the stimulation threshold of the facial nerve was measured in miliamper (mA) units. After crushing the nerve, the stimulation threshold of the facial nerve was re-measured, and the wound was closed in a single layer with 4-0 vicryl (Ethicon, Germany). After 30 days of ozone therapy, the facial nerves in the rats were once again exposed, and the nerve stimulation thresholds were measured. The results were compared with those of the control group.

### Pathological evaluation

#### Light microscopic assessment

The left facial nerve was dissected from the adjacent tissues after measuring nerve stimulation thresholds, and the crushed part of the facial nerve was then excised. All specimens were fixed in 10% formaldehyde with tamponade. After fixation, specimens were embedded in paraffin blocks, and 4 μm sections were collected. All specimens were stained with hematoxylin-eosin and toluidine blue, and were examined by a pathologist via light microscopy. All specimens were investigated for the degree of macrovacuolization, vascular congestion, and myelin sheath thickness. Macrovacuolization and vascular congestion were graded as none, mild, moderate, or severe. Thickness of the axonal myelin sheath was categorized as very thin, thin, or normal.

#### Electron microscopic assessment

Excised facial nerves were fixed in 2.5% glutaraldehyde (Electron Microscopy Sciences, Fort Washington, PA, USA), postfixed in 1% osmium tetroxide (Electron Microscopy Sciences) and processed routinely for electron microscopy and embedded in resin (Electron Microscopy Sciences). Ultra-thin sections (50–70 nm) were cut with an ultramicrotome (Leica Microsystems GmbH, Wien, Austria), contrasted with uranyl acetate-lead citrate, and examined with an electron microscope (JEOL-JEM 1011, Jeol Ltd., Tokyo, Japan). Samples were photographed with a digital camera (Megaview III, Olympus Soft Imaging Solutions GmbH, Münster, Germany) attached to the microscope.

Previously described grading systems were used for the ultrastructural evaluation of myelin sheaths and axons damage in the nerve fibers.^10^, ^11^ Myelinated axons were graded ultrastructurally as grade 0 (normal), grade 1 (separation in myelin configuration), grade 2 (interruption in myelin configuration), grade 3 (honeycomb appearance), or grade 4 (collapsed myelin forming ovoids). Axonal ultrastructure was scored according to damage as 0 (no damage) 1+ (low damage), 2+ (mild damage), or 3+ (high damage). Seven samples from each group were analyzed by this quantitative evaluation. During these grading procedures, 50 myelinated axons from each sample were evaluated.

### Statistical analysis

Statistical analyses of the data were conducted using SPSS ver. 15.0. Nonparametric Wilcoxon signed-rank test was used for the comparison of two dependent groups. Nonparametric Mann–Whitney *U* test was used for the comparison of independent groups. Student's *t*-tests were used for the evaluation of electron microscopy. *p*-Values <0.05 were considered statistically significant.

## Results

There was no statistically significant difference in stimulation thresholds between the ozone and saline groups after crushing (*p* = 1.000), indicating that the severity of the nerve crush injury was similar in both groups. Although stimulation thresholds were significantly lower from pre-crush thresholds in both the ozone and saline groups (*p* = 0.018 and 0.018) after 30 days of treatment, the ozone-treated group had lower stimulation thresholds than the saline group when compared to post-crushing levels. Although this did not reach the level of statistical significance, it indicates greater functional improvement in the ozone group (*p* = 0.053). Improvement was also seen in the saline group but this was thought to be a result of spontaneous recovery of the facial nerve. After 30 days of treatment, neither group reached pre-crushing amplitude levels (Table 1).

**Table 1:** Comparison of facial nerve stimulation thresholds before and after crushing and 30 days later.

	Before crush (mA)		After crush (mA)		30 days later (mA)		Comparing to before crush and 30 days later	Comparing to after crush and 30 days later
	Mean (SD)	Median	Mean (SD)	Median	Mean (SD)	Median	*p*^a^	*p*^a^
Control	0.068 (0.017)	0.061	0.935 (0.203)	0.903	0.099 (0.038)	0.084	0.176^b^	0.018^b^
Ozone	0.052 (0.127)	0.053	0.925 (0.127)	0.904	0.067 (0.011)	0.064	0.090^b^	0.018^b^
*p*^c^	0.165		1.000		0.053			

a Wilcoxon signed-rank test.

b *p* < 0.05.

c Mann–Whitney *U* test.

**Figure 2:**
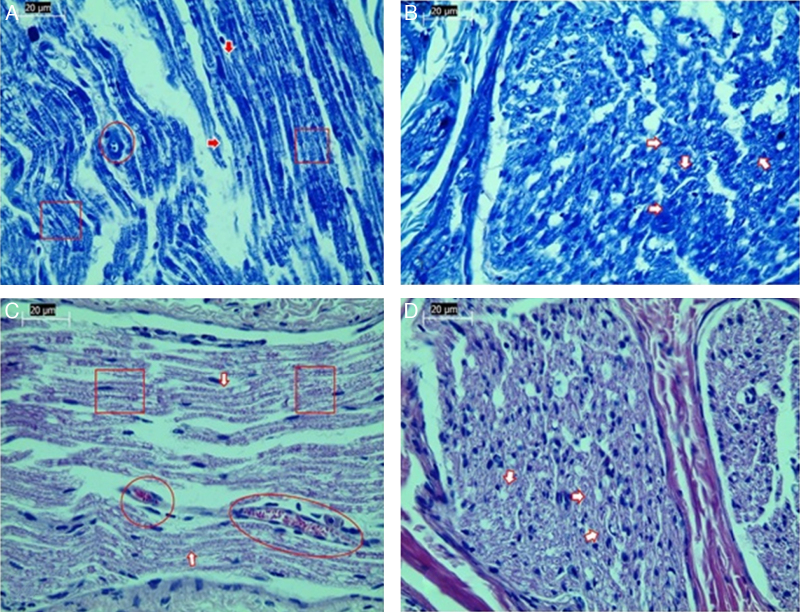
Examination of axonal structure post-injury by light microscopy. (A) Toluidine blue staining of control group. (B) Toluidine blue staining of ozone group. (C) H&E staining of control group. (D) H&E staining of ozone group. Magnification was 40×; arrows indicate myelin sheath, circles indicate areas of vascular congestion, and squares indicate macrovacuolization. Representative images are shown.

**Figure 3:**
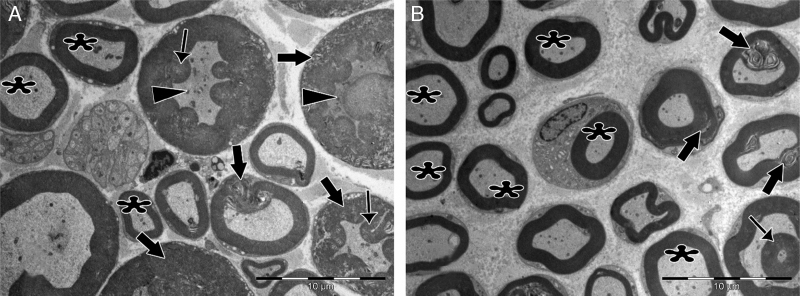
Transmission electron micrographs of myelinated axons post-injury. (A) In the control group, delamination and deformation were seen in myelin sheaths of most of the axons (bold arrows), in addition to the appearance of myelin ovoid bodies (thin arrows) and darkened axonal cytoplasm (arrowheads). Very few normal myelinated axons (asterisks) were seen. (B) In the experimental group, numerous normally myelinated axons are seen (asterisks). Delamination and deformation in myelin sheaths (bold arrows) and myelin ovoid bodies (thin arrow) are shown in only a few myelinated axons. Magnification for both images: 4000×, representative images are shown.

**Figure 4:**
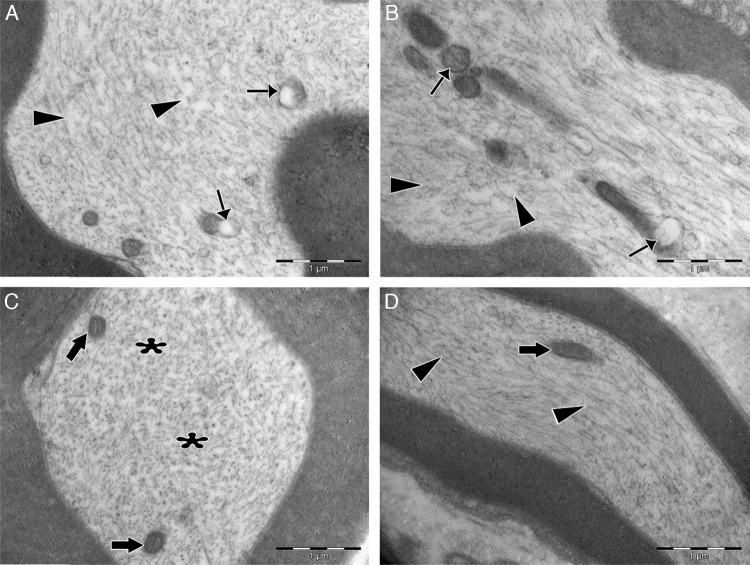
Transmission electron micrographs of cytoplasm of myelinated axons. (A) In the control group, swollen mitochondria with degenerated cristae structure (thin arrows) were seen in the cytoplasm. Microtubules and microfilaments were dispersed heterogeneously. In some areas, a decreased amount of microtubules and microfilaments was observed (arrowheads). (B) In the control group, swollen and damaged mitochondria were seen (thin arrows), and microtubule and microfilament arrays were disorganized and disrupted (arrowheads). (C) In the experimental group, the ultrastructure of mitochondria was normal (bold arrows). Microtubules and microfilaments were dispersed homogenously throughout the cytoplasm (asterisks). (D) In the experimental group, longitudinally aligned microtubules and microfilaments were normal (arrowheads), and mitochondria exhibited normal ultrastructure (bold arrows). Magnification for all images: 3000×, A and C are cross sectional views, B and D are longitudinal sections, and representative images are shown.

Significant differences were found in vascular congestion, macrovacuolization, and myelin thickness between the ozone and control groups by light microscopy (Table 2; Fig. 2A–D). Severe degeneration to different degrees was observed in almost all of the myelinated axons in the control group by electron microscopy. In slightly damaged myelinated axons, while the myelin sheaths had mild delamination, the cytoplasmic structure of the myelinated axons was normal. In severely damaged myelinated axons, severe delamination and disintegration were observed in myelin sheaths. Mitochondrial swelling and loss of cristae, and disorganization of microtubules and microfilaments were observed in the axonal cytoplasm, In addition, some axons were darkened and contained myelin ovoid bodies (Figure 3, Figure 4).

**Table 2:** Comparison of histopathology variations in the facial nerve between ozone and saline groups after 30 days via light microscope.

	Control (*n* = 7)	Ozone (*n* = 7)	
	*n* (%)	*n* (%)	*p*
*Vascular congestion*			0.017^a^
None	0 (0)	3 (42.8)	
Mild	2 (28.6)	3 (42.8)	
Moderate	2 (28.6)	1 (14.3)	
Severe	3 (42.8)	0 (0)	
Median	2	1	

*Macrovacuolization*			0.002^b^
None	0 (0)	2 (28.6)	
Mild	0 (0)	4 (57.1)	
Moderate	5 (71.4)	1 (14.3)	
Severe	2 (28.6)	0 (0)	
Median	2	1	

*Myelin thickness*			0.053
Very thin	6 (85.7)	2 (28.6)	
Thin	1 (14.3)	2 (28.6)	
Normal	0 (0)	3 (42.8)	
Median	2	1	

Mann–Whitney *U* test.

a *p* < 0.05.

b *p* < 0.01.

In the ozone group, most of the myelinated axons were normal in structure under electron microscopy. Mitochondrial ultrastructure and organization of microtubules and microfilaments in axonal cytoplasm were normal in myelinated axons. However, in a few of the myelinated axons, separation of the myelin configuration and disintegration of myelin sheaths was observed. Furthermore, there were swollen mitochondria with damaged cristae, disorganized microtubules, and microfilaments and myelin ovoid bodies in the myelinated axon cytoplasm (Table 3; Figure 3, Figure 4).

**Table 3:** Comparison of histopathology variations in the facial nerve between ozone and saline groups after 30 days via electron microscope.

	Control (*n* = 7)	Ozone (*n* = 7)	
	Mean (SD)	Mean (SD)	*p*^a^
*Ultrastructural grading of myelinated axons*			
Grade 0	8.71 (3.039)	23.00 (6.856)	0.001^b^
Grade 1	12.00 (4.041)	13.71 (2.984)	0.386
Grade 2	10.86 (3.078)	5.71 (2.138)	0.004^b^
Grade 3	10.00 (3.830)	3.29 (2.563)	0.003^b^
Grade 4	9.00 (1.414)	4.29 (2.215)	0.001^b^

*Damage to axonal ultrastructure*			
0	11.43 (3.309)	24.00 (3.830)	0.000^b^
1+	10.71 (4.536)	15.57 (2.149)	0.032^c^
2+	13.43 (3.259)	5.57 (1.813)	0.000^b^
3+	14.43 (3.735)	4.86 (3.338)	0.000^b^

a Student's *t*-test.

b *p* < 0.01.

c *p* < 0.05.

## Discussion

Many drugs are used in the treatment of traumatic facial paralysis. The most commonly used is corticosterone, which decreases capillary permeability and reduces edema around the facial nerve. Corticosterone is also thought to decrease degeneration of the axon while increasing regeneration.^2^, ^12^, ^13^, ^14^ The effect of vitamin E on nerve regeneration after trauma has also been investigated.^15^ Vitamin E is a powerful fat-soluble antioxidant, which can prevent formation of free radicals, protecting cells against oxidative stress and lipid peroxidation. Taskale et al.^15^ investigated the effects of vitamin E and vitamin E plus corticosterone on facial nerve healing in rats. They found that vitamin E had a positive effect on nerve healing; this effect was enhanced by the addition of corticosterone. Lieberman et al.^16^ investigated the effects of corticosteroids on functional recovery and neuron survival after facial nerve injury in mice. They found that corticosteroid treatment slows functional recovery and disturbs neuron survival following facial nerve crush injury in adult mice. They also claimed that the degree of motor neuron survival corresponds with functional status. In juvenile mice, crush injury results in overall poor functional recovery and profound cell loss in the facial motor nucleus. In another study, Toros et al.^17^ evaluated the effects of Hyperbaric Oxygen (HBO), methylprednisolone and combined HBO–methylprednisolone treatments on traumatic facial nerve regeneration in rats. HBO is thought to decrease injury-related edema, decrease the concentration of oxygen free radicals after ischemia with reperfusion and increase local tissue oxygen levels.^18^ They concluded that combination therapy with methylprednisolone and HBO might be beneficial for treating the ischemia and edema that both result from the facial nerve injury cascade.

Spontaneous nerve regeneration with good functional improvement can be found after crush injury of peripheral nerve.^19^ This kind of nerve injury is treated with pharmacological agents, instead of surgery. The healing process after crush injury is largely impaired due to increased production of free radicals, rather than neuroinflammation and edema.^20^ Antioxidant materials scavenge free radicals and contribute to nerve regeneration. Antioxidant enzymes, such as superoxide dismutase and catalase, protect cells from the toxic effects of free radicals. Free radicals induce traumatic cell damage causing cell death. Crush injury to nerves leads to oxidative stress, such as lipid peroxidation of neurovascular cells, by creating free radicals.^21^, ^22^

A number of antioxidants that have been examined for their ability to improve regeneration of peripheral nerves. Jang et al.^23^ investigated the effect of ginkgo biloba extract on recovery after facial nerve crush injury in the rat. They found that the intraperitoneal injection of ginkgo biloba extract was effective in promoting regeneration of the nerve in an experimental facial nerve crush rat model. Another antioxidant that has been studied for use in supporting the regeneration of crushed facial nerve is coenzyme Q.^24^ Coenzyme Q was also found to be effective in promoting the regeneration of the nerve in an experimental facial nerve crush rat model.

Ozone may promote a moderate oxidative stress that, in turn, increases endogenous antioxidant systems.^25^, ^26^ The protective mechanism mediated by ozone may involve protein synthesis. Increased reactive oxygen species can induce antioxidant gene expression in many cells. A major mechanism of redox homeostasis is reactive oxygen species-mediated induction of signal cascades that increase expression of antioxidants.^27^ Therefore, since ozone increases antioxidant levels, leading to a decrease in free radicals, we investigated the effect of ozone therapy on the regeneration of crushed facial nerves.

In the current study, regeneration of the facial nerve was evaluated by assessing electrophysiological thresholds and by histopathological examination. There are a number of nerve integrity monitoring devices available to identify and preclude persistent nerve damage.^28^, ^29^ In this study, we used the Nerve Integrity Monitor facial electromyography technique described by Delgado et al.^29^ and used in several previous studies^15^, ^24^ to record the contraction of facial muscles. In the present study, although not reaching statistical significance, there was improvement in facial nerve function in the ozone group when assessed using threshold levels. There are several studies in the literature reporting histopathological evaluation of the degree of macrovacuolization, vascular congestion, and myelin sheath thickness to assess nerve damage.^17^, ^24^ In the present study, we used similar parameters to assess nerve structure; significant differences were found in vascular congestion, macrovacuolization, and myelin thickness between the ozone and control groups.

The strength of our study was in the evaluation of the degree of facial nerve regeneration not only by light microscope, but also by electron microscopy. A large number of myelinated axons from each sample (*n* = 50) and a total of 350 myelinated axons from each treatment group were evaluated by electron microscopy. In this study, electron microscopy confirmed the findings of light microscopy. A limitation of our study was the short duration of follow-up after the treatments. We explored facial nerves 1 month post-treatment. This would need to have been much longer in order to have observed functional recovery. This may be one of the reasons our findings did not reach statistical significance. Therefore, further studies are needed with longer follow-up after treatment. We hope that this preliminary study will encourage other larger studies to be undertaken.

## Conclusion

In conclusion, our study is the first of which we are aware to investigate the effects of ozone therapy on the regeneration of crushed facial nerves. Our data suggests that ozone therapy may have beneficial effects on the regeneration of crushed facial nerves. Its positive effects were seen especially on the pathologic evaluation. Electron microscopy confirmed the results of light microscopy. Therefore, we conclude that ozone therapy may be a promising avenue to explore for the treatment of acute facial paralysis and peripheral nerve regeneration. Further studies are needed to confirm our findings.

## Conflicts of interest

The authors declare no conflicts of interest.
